# Dental Education

**Published:** 1900-07

**Authors:** Eugene S. Talbot

**Affiliations:** Chicago, Ill.


### DENTAL EDUCATION.[1]

BY DR. EUGENE S. TALBOT, CHICAGO, ILL.

Mr. President and Members of the American Academy of Dental Science,—I am sure you will agree that it is high time that dental education was placed upon a broader basis than now exists. It is admitted that, when the brain is concentrated upon one department of science and severed from others, it is narrowed and the important matters are neglected. This is true particularly of dentistry. The establishment of distinct dental colleges in 1840 began the narrowing of our calling. From year to year this narrowing has been going on until, to-day, we do not know whether we are a trade or a profession. Students in dental colleges look upon anatomy, physiology, materia medica, and chemistry as fads distinct from filling cavities of the teeth and crown- and bridge-work,

[1] Read before the American Academy of Dental Science, April 4, 1900.

the central notions taught in dental colleges. The tendency to narrow specialism is strong, and, together with commercialism, narrows the profession from year to year.

The National School of Technique is the last straw upon the breaking camel's back. The central idea of preparation of cavities and filling teeth has been pushed to its extreme limit. "Measurement of cavities" and cutting away hard, sound, healthy tissue on approximal surfaces, while neglecting to remove the cause of decay, is, essentially, removing arm or leg because finger or toe is diseased.

A bill is now before Congress for the appointment of dentists in the army and the navy. Should this bill become a law, what dentist would be qualified to go to the Philippines and cope with the diseases of the mouth and jaws from which the soldiers are now suffering? To be sure, he could measure cavities, cut away good, sound tissue, and fill teeth; but what scientific status could such a man have?

Dental literature and dental societies of to-day exhibit marked evidences of lack of broad scientific principles. The jaws and teeth are intimately related parts of the general organism. Diseases which affect the body or an organ also affect the jaws and teeth, and decayed teeth and diseases of the jaws affect the general economy as a whole. With all the skill of dental mechanics, dentists are unable to arrest decay, nor will they be by the present methods of practice. Decay of the teeth is ever on the increase. Departments of medicine have already taken steps to inquire into the cause.

Interstitial gingivitis, or so-called pyorrhœa alveolaris, is more to be dreaded than decay of the teeth. Since eighty per cent. of this disease is due to systemic causes which cannot be reached by local treatment, it is no wonder that the profession are at sea as to causes and ultimate results.

The first paper upon irregularities of the teeth was written in the year 1796, yet one hundred and four years after, a professor in two dental colleges claims that it is a subject for to-morrow rather than to-day. Another professor indulges in the charlatan-like cant that it is enough for him to know how to treat the deformity, and he cares nothing for the cause. Enough, however, has been said to show that broader medical training is necessary if patients are to be treated in an intelligent manner.

There was *once* a field for narrow dental-college teaching. Such teaching in its narrow limits, however, soon becomes deteriorating. The tendency to overtreat and overdo certain operations is now well established. Two decades ago I expressed the opinion, to which I still more emphatically adhere, that if progress was to be made in dentistry, it must be along the lines of a broad medical education.

The country is full of inferior dental colleges graduating students unfitted for practice. Better schools are kept at a lower level by the National Board of Dental Faculties because of these schools. Why such universities as Harvard, University of Pennsylvania, University of Michigan, Northwestern University, and others standing high in educational matters otherwise, allow the dental departments to fall so low is beyond my conjecture.

"The government of Belgium is considering a bill advanced by the majority of physicians to suppress the diploma of dentistry and only allow the practice of dentistry to qualified physicians as a branch of the medical sciences, like laryngology, ophthalmology, etc. Beco, the chairman of the special committee, enumerates, among the reasons for this step, the overcrowding of the medical profession and the necessity for considering dentistry as an important and lucrative specialty in the domain of general medicine. The standard that has hitherto been required of dentists has been so low that some change is imperative, and suppression of special diplomas to dentists seems the simplest and most practical solution of the question under the present circumstances." [1]

"A memorial has been presented to the Board of Management of the London Hospital, signed by about four-and-twenty gentlemen who hold, or have held, the office of dental surgeon to metropolitan general hospitals, urging that all candidates for these appointments should be required to be registered both upon the Medical and Dental Registers. There seems to be several good reasons why such a course should be adopted; hospitals require of their surgeons the possession of the Fellowship (which is, as a matter of fact, possessed by several of the dental surgeons), and there seems no reason why, in the case of a dental surgeon, they should be content with the license in dental surgery, which is the minimum qualification which entitles a man to practise dentistry. Then, again, he is concerned much more with the teaching of medical than of dental students, and he will not alone be in a better posi-

---

[1] Journal of the American Medical Association, February 17, 1900.

tion to avail himself of cases which lie on the border between surgery and dentistry, but he will teach with more authority and influence if he be qualified in medicine and surgery. The memorial, therefore, appears to be well timed and its arguments well grounded. It will, without doubt, have much weight with the body to which it is addressed." [1]

Dr. E. A. Bogue informs me that to practise dentistry in Austria one must be a graduate of medicine.

It will be seen, therefore, that there is a general feeling that the dentist should be medically educated. There are many professors and teachers, even in our university dental departments, who say that they do not believe that it is necessary for the dentist to be medically educated to practise his profession. This feeling, no doubt, is due to the fact that they have been educated along these narrow lines. Their lack of knowledge of general pathology demonstrated by their writings is a standing witness of this deficiency.

When the subject of a medical education is suggested for dentists, the old hackneyed reply is always made that too much time and money are required for the dentist to take both a medical and a dental course. Lately it has been claimed, moreover, that lengthening of the medical course of study and the necessary laboratory work make it impossible for the dentist to cover both medicine and dentistry.

Dr. N. S. Davis, the father of the American Medical Association, president of the Ninth International Medical Congress, settled these questions more than three decades ago. In responding to the sentiment offered by Dr. C. W. Spaulding, then president of the American Dental Association,—" To the president of the American Medical Association; Medicine, Surgery, and Dentistry, departments of a common science; their disciples should constitute a common brotherhood,"[2] he went over the entire field, and showed that dentistry was as much a part of general medicine as ophthalmology, otology, etc., and that dentistry should be studied as a part of general medicine. His idea was at that time, and is to-day, that all students should matriculate in the medical department and study the general branches of medicine together for a certain number of years, after which the students should take up the hospital and

---

[1] British Medical Journal, January 6, 1900.

[2] Chicago Medical Examiner, September, 1865, page 576.

laboratory work which fits them for the special department of medicine which they expect to follow. The plan is perfectly simple, possible, and the expense to the university is greatly diminished. The details, of course, must be worked out by the universities. President Eliot has suggested a similar plan, which will cover all the difficulties that stand in the way of broad medical training,—that of a "university of medicine which shall teach all that has to do with animal life in health and sickness." When this is accomplished, dentistry, with its almost perfect mechanical skill, will scientifically equal any other department of medicine. Presidents and boards of regents of universities having dental departments should nourish and strengthen this youngest and most feeble offspring of medicine until it is as robust as the other departments. When this is accomplished, scientists, medical men, and dentists abroad will look upon American dentistry as a science.

